# Collaborators

**Published:** 1886-01-20

**Authors:** 


### EDITORIALS AND MISCELLANEOUS.

### COLLABORATORS.

We have secured the services of a few intelligent and able writers as collaborators in the original department of our journal for the present year, and to these others will be added hereafter.

In seeking to give, as we do, gleanings from the entire field of Medical Literature, the number of items in each copy is so great that we find there is not sufficient space for the table of Contents on the first page of the cover; hence we have been compelled to transfer this table to the second page of the same.

Our RECORD is prospering, and numerous testimonials could be given from the correspondence of the office to show that it is the favorite of the busy practitioner in the Southern and Western States. So, with much encouragement and renewed energy, we enter upon the labors of 1886, and extend to our numerous readers and friends the greetings of the season.

---

THE American Medical Association is appointed to meet in St. Louis, Mo., the first Tuesday in May, 1886.

